# Partial Sacral Resection for the Treatment of Isolated Testicular Tumor Metastasis

**DOI:** 10.7759/cureus.34618

**Published:** 2023-02-04

**Authors:** Hiroto Kamoda, Toshinori Tsukanishi, Hideyuki Kinoshita, Tsukasa Yonemoto, Takeshi Ishii

**Affiliations:** 1 Orthopedic Surgery, Chiba Cancer Center, Chiba, JPN; 2 Orthopedic Surgery, Tokyo Medical University Ibaraki Medical Center, Ami, JPN; 3 Orthopaedic Surgery, Chiba Cancer Center, Chiba, JPN

**Keywords:** combined anterior and posterior surgery, isolated metastasis, pet-ct, nonseminoma, partial sacrectomy

## Abstract

We encountered an uncommon case of a non-seminomatous germ cell tumor with solitary bone metastasis at the initial presentation. A 30-year-old male patient with testicular cancer underwent an orchidectomy and was diagnosed with non-seminoma. Positron emission tomography-computed tomography detected an isolated metastatic lesion in the right sacral wing, which disappeared after a series of chemotherapy. En-bloc surgical resection was performed as curative local treatment, and the patient was able to perform his activities of daily living with no apparent recurrence. Therefore, this surgical method is considered safe and beneficial for the treatment of sacral wing lesions.

## Introduction

Testicular cancer is the most common solid-organ cancer diagnosed in adult men under the age of 34 years [1]. Most patients with testicular cancer are diagnosed early and at a localized stage [2]; however, 12% of diagnosed cases were stage III, with distant metastasis at the initial visit [3]. In testicular germ cell tumors (GCTs), bone metastasis is usually observed in advanced stages and is accompanied by metastasis to other sites, such as the lymph nodes or lungs. Nonseminomatous GCTs (NSGCTs) with isolated bone metastases have been described as exceptionally rare [4]. According to the National Comprehensive Cancer Network (NCCN) [A1] guidelines, nonseminomas occurring after the primary chemotherapy are treated only if the residual disease is found and serum tumor markers alpha-fetoprotein (AFP) and beta-human chorionic gonadotropin (HCGβ) have been normalized, so that all sites of residual disease can be resected. Here, we present a case of NSGCT with isolated bone metastasis initially detected by positron emission tomography-computed tomography (PET-CT).

## Case presentation

**Figure 1:**
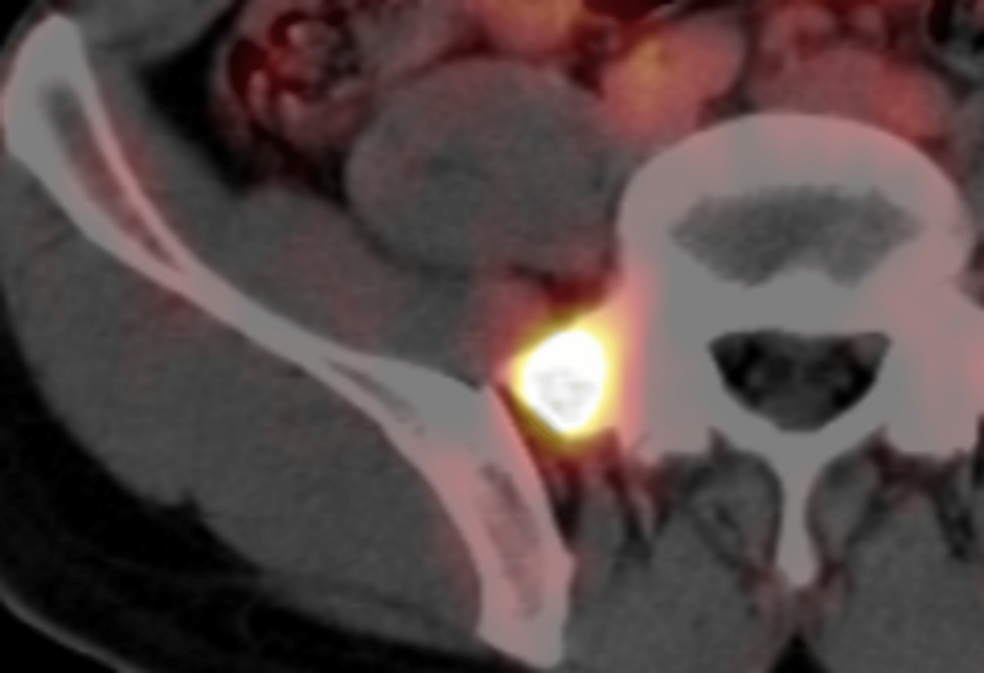
18F-FDG PET/CT imaging before the chemotherapy

A 30-year-old man consulted a nearby doctor due to the occurrence of swelling and pain in his right testis. He was diagnosed with epididymitis. This pain improved once the medication was administered, but symptoms recurred after two weeks. By palpation by the same nearby doctor, a solid mass was detected in his right testis, and AFP and lactic acid dehydrogenase (LDH) values were elevated and slightly elevated (87.4 and 320, respectively), whereas HCG and HCGβ were within the normal range. He was diagnosed with testicular cancer and underwent a right orchidectomy. At that time, no obvious metastasis was observed. From the pathological results of the extracted specimen, he was diagnosed with “non-seminoma” stage IA (TNM: pT1N0M0), because the tumor was locally invasive without evidence of distant metastases. After the test, tumor maker values improved (AFP=3.44, LDH=163). However, to reduce the risk of recurrence, the patient was referred to our hospital for postoperative chemotherapy. Before chemotherapy, ^18^F-FDG PET/CT(PET-CT) was performed, which showed an accumulation in the right sacral wing (Figure 1). No accumulation was observed except in the sacrum.

**Figure 2:**
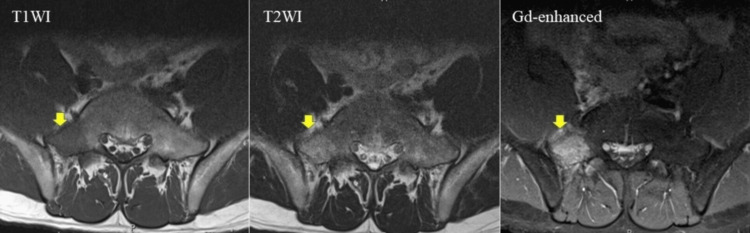
MRI before chemotherapy MRI showed a T1 low signal, T2 high signal and Gd contrast effect on the right side of the sacrum.

Magnetic resonance imaging revealed that the right sacral wing showed hypointense signals on T1-weighted imaging (T1WI), hyperintense signals on T2WI, and a clear Gd-contrast effect (Figure 2).

**Figure 3:**
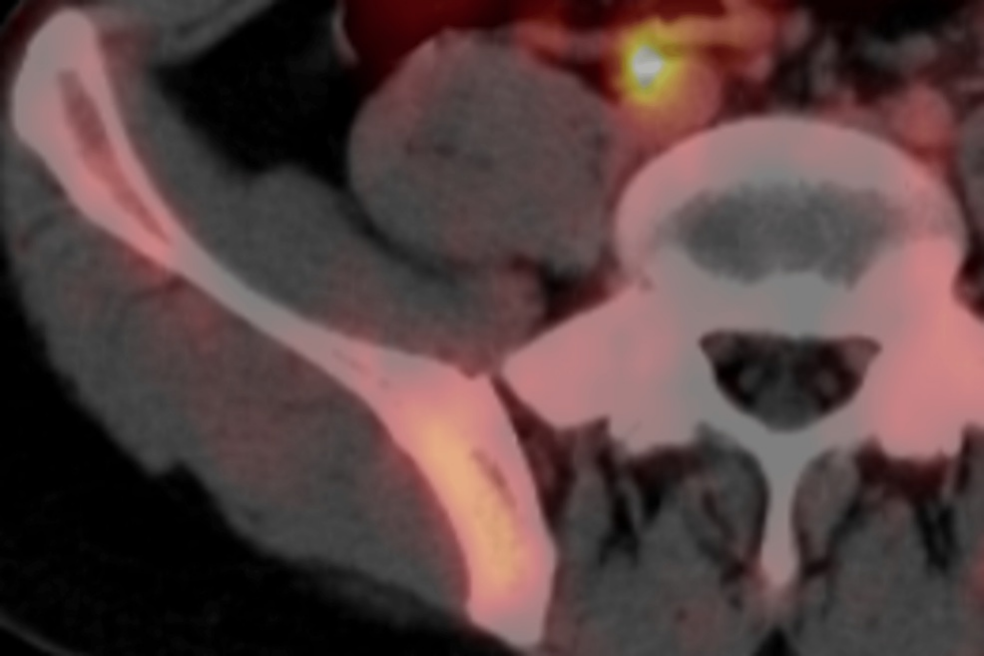
PET-CT after the chemotherapy

One week after the PET-CT imaging, a needle biopsy was performed, and the pathological diagnosis was testicular cancer metastasis. At this point, his tumor grade was changed to “stage IIIC.” Based on the tumor board, four cycles of etoposide, ifosfamide, and cisplatin (VIP) were administered at three-week intervals. After chemotherapy, a repeat PET-CT was performed to determine treatment effects. On examination, the sacral wing accumulation disappeared. Therefore, the series of chemotherapy regimens were considered effective (Figure 3).

In response to this result, local treatment was planned for definitive treatment. As a result of an in-hospital meeting, surgical resection was judged to be preferable over carbon-ion radiotherapy.

**Figure 4:**
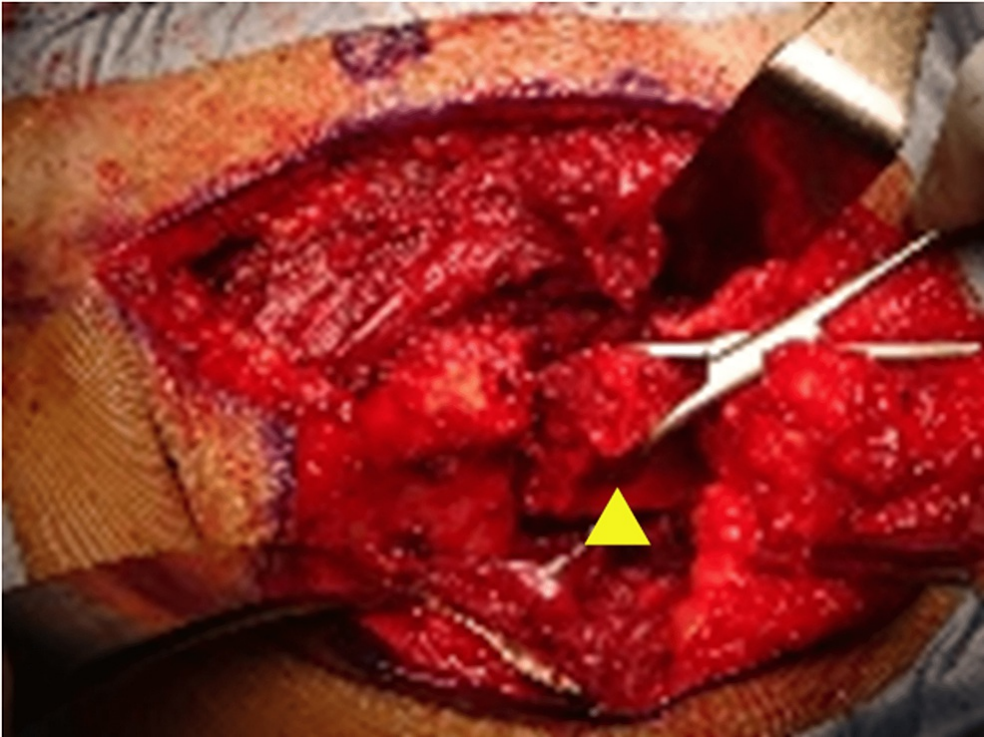
Surgical view from behind The sacral wing lesion (▲) was removed as a single mass.

Surgery was performed under general anesthesia with the assistance of abdominal surgeons. First, the peritoneum was exposed through a midline abdominal incision. After the peritoneal incision, the intestine was protected, and the anterior surface of the middle sacrum and its right-wing were exposed. The common iliac artery and vein were protected, and a 5-mm depth gutter was made along the anterior sacral foramina in the right front of the sacrum. Next, a 10-cm-wide posterior part of the right ilium was removed from behind to expose the posterolateral side of the sacral wing. A laminectomy of the sacrum was performed to identify the cauda equina and right sacral nerve roots. After confirmation of the safety of the nerve roots, a bone incision was made from behind the sacrum toward the pre-reamed anterior osteotomy line to complete en-bloc resection of the sacral wing lesion (Figure 4). Because of the possibility of additional chemotherapy after surgery, internal fixation was not used to avoid infection as much as possible.

**Figure 5:**
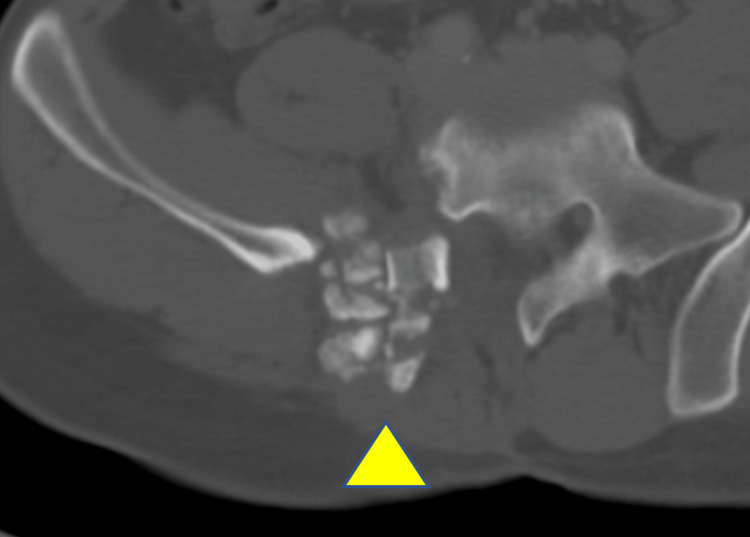
CT imaging after surgery Right sacral wing was resected and the grafted bones were placed (▲) at the same site.

The resected right ilium was used as graft bone in the right sacral wing defect (Figure 5).

The operative time was 648 min, and the bleeding volume was 1,680 mL. The pathological report indicated that the final diagnosis of the surgical specimen was non-seminoma, and tumor cells were found to be mostly necrotic. The postoperative process was going well, and dietary intake was initiated two days postoperatively. Ambulatory training was initiated seven days postoperatively. He could ambulate with a cane 10 days postoperatively and was discharged from the hospital. At the two-year follow-up, he did not experience any symptoms at the surgical site. He could ambulate without a cane, and no apparent recurrence was observed.

## Discussion

Germ cell cancer is the most common malignancy in men aged 15-40 years, and the majority of patients present with a primary tumor in the testis [5]. Primary orchidectomy is the primary treatment choice for testicular tumors to determine the pathological diagnosis of seminoma or non-seminoma. Metastatic lesions are frequently observed in the retroperitoneal lymph nodes or lungs, and metastatic bone disease is relatively uncommon [6]. In 1997, the International Germ Cell Cancer Collaborative Group [A1] published a prognostic classification system. Patients with bone metastases are classified in “Poor prognosis” group, with an estimated 5-year survival rate of 67% according to the 2020 UPDATE edition. In some previous reports, the incidence of bone metastasis in testicular tumors was 1%-3% at presentation [7,8]. Jamal-Hanjani et al. reviewed 2,550 patients with GCTs, and 19 of them had bone metastasis. Metastasis was commonly seen in the vertebrae (79%), pelvis (16%), ribs (16%), and femur (11%). Bone metastasis was diagnosed based on symptoms in 37% of patients but on incidental findings on imaging in 58% of all patients [9]. It is difficult to determine the presence of bone metastases by blood tests since tumor markers, namely AFP, HCG, and LDH, did not differ between patients with or without primary bone metastases [10].

Biebighauser et al. described that NSGCT metastasis usually occurs via the lymphatic vessels, spreading first to retroperitoneal lymph nodes and then sequentially to extranodal sites [4]. Moreover, some reports demonstrated that hematogenous metastasis through the general circulation was the most frequent route of bone metastasis in patients with testicular cancer [7,11-13]. However, when isolated metastasis in the vertebrae or ribs was observed without lung or liver metastasis, vertebral vein plexus metastasis, called Batson’s plexus, was considered [11,12]. We inferred that the sacral metastasis in this case occurred through this plexus.

PET-CT has been used to detect metastatic lesions in patients with various cancers. For patients with testicular cancer, PET-CT is helpful when primary staging CT scans are equivocal but insufficiently sensitive to predict relapse [14]; however, data available on early-stage tumors are minimal [15]. In our case, it was not possible to identify bone metastasis without PET-CT due to the absence of subjective symptoms. Therefore, this study indicates that PET-CT may be necessary for detecting metastatic bone lesions and primary staging.

Based on PET-CT and sacral needle biopsy, this case was classified as stage IIIC according to the 2016 NCCN guidelines. In this guideline, if the tumor remains without an increase in tumor markers, the remaining tumors are recommended to be resected surgically. In our case, the accumulation of the right sacral wing on PET-CT disappeared after the first-line chemotherapy. At the time, sacral wing resection was planned considering the risk that the tumor or teratoma components remained. To date, reports on sacral wing resection have been limited. Altan et al. reported partial sacrectomy and iliac wing resection for a brown tumor in the sacral vertebrae due to parathyroid adenoma [16]. However, a detailed surgical method has not yet been described.

In our case, the surgical procedure was initially performed using a transabdominal approach. With this approach, the intestine and common iliac vessels were securely protected, and the anterior surface of the middle sacrum and its right wing was well exposed. Therefore, subsequent sacral wing dissection and extraction from the back were completed without concern about organ injury. We believe that this procedure is suitable for patients with bone metastasis confined to the sacral wing, but it still has a disadvantage; the normal posterior iliac region should be widely resected, as was performed in this case. Recently, the use of navigation systems has begun to spread in the assistance of orthopedic surgery, mainly spinal surgery. We did not have a navigation system; however, the ilium could have been preserved had we used a navigation system and performed the sacral osteotomy with real-time imaging rather than under direct vision.

## Conclusions

In conclusion, we encountered a case of sacral wing metastasis originating from a testicular tumor. PET-CT was essential for the detection of the metastatic lesion, and en-bloc sacral wing resection was safely performed using both anterior and posterior approaches. Our surgical method was considered useful as a radical treatment for isolated metastasis of the sacral wing.
